# Nuclear Distribution of RNA Polymerase II and mRNA Processing Machinery in Early Mammalian Embryos

**DOI:** 10.1155/2014/681596

**Published:** 2014-04-29

**Authors:** Irina O. Bogolyubova, Dmitry S. Bogolyubov

**Affiliations:** Laboratory of Cell Morphology, Institute of Cytology RAS, 4 Tikhoretsky Avenue, St. Petersburg 194064, Russia

## Abstract

Spatial distribution of components of nuclear metabolism provides a significant impact on regulation of the processes of gene expression. While distribution of the key nuclear antigens and their association with the defined nuclear domains were thoroughly traced in mammalian somatic cells, similar data for the preimplantation embryos are scanty and fragmental. However, the period of cleavage is characterized by the most drastic and dynamic nuclear reorganizations accompanying zygotic gene activation. In this minireview, we try to summarize the results of studies concerning distribution of major factors involved in RNA polymerase II-dependent transcription, pre-mRNA splicing mRNA export that have been carried out on early embryos of mammals.

## 1. Introduction

The main feature of the eukaryotic cell is the nucleus that holds the genetic information and realizes DNA replication and the processes of gene expression. Being the place where transcription and the related events occur, the nucleus demarcates nuclear processes from translation in cytoplasm, providing new additional levels of the regulation of gene expression that cannot be realized in prokaryotes.

Interchromatin space of the nucleus contains different nuclear domains. In mammalian somatic cells, molecular composition of major functional nuclear domains has been studied in detail [1]. Initially, the nuclear distribution of pre-mRNA splicing factors and the structures enriched with these factors have been explored with the use of antibodies against the Sm antigen (now known as symmetric dimethylarginine, sDMA) [2], 2,2,7-trimethylguanosine (TMG) cap of snRNAs, and some “signature” proteins of nuclear domains (coilin, SR-proteins) [3]. As a result, interchromatin granule clusters (IGCs), known as nuclear splicing speckles in terms of fluorescent microscopy, and also Cajal bodies have attracted a special attention as evolutionary conserved and “universal” nuclear domains [4].

IGCs/speckles are nuclear domains enriched in pre-mRNA splicing factors (snRNPs and SR-proteins), located in the interchromatin space of the nucleus. These domains are dynamic nuclear organelles, and their constituents can exchange continuously with the nucleoplasm and other nuclear locations, including active transcription sites [5]. IGCs not only serve as transient reservoirs for pre-mRNA splicing factors, but are also involved in many other functions (for reviews, see [5, 6]). The Cajal bodies have been implicated in RNA-related metabolic processes such as snRNP biogenesis, maturation and recycling, histone mRNA processing, and telomere maintenance (for reviews, see [7, 8]). Deciphering the fundamental rules that impact nuclear functions in the whole seems to be impossible without a large-scale comparative approach. As compared with traditionally used experimental objects including tissue-culture somatic cells, early mammalian embryos have been explored with a lesser extent.

The nuclei of early mammalian embryos are characterized not only by the unique functional status, but also by a peculiar nuclear ultrastructure [9]. Stepwise physiological reactivation of chromosomal transcription activity during early embryogenesis, known in literature as zygotic gene activation (ZGA), suggests that the embryos could be attractive experimental models in order to analyze nuclear distribution of gene expression factors that virtually may associate with the universal and/or specific nuclear domains. ZGA is one of the key points of the maternal-to-zygotic transition (MZT) that involves a set of structural, molecular, and biochemical rearrangements [10–14]. However, the majority of studies concerned first of all the changes of gene expression patterns [15–17] as well as the processes of chromatin remodeling during realization of ZGA events (for reviews, see [18, 19]). The relationships between these processes and the distribution of the essential components of nuclear metabolism were described with a lesser extent.

Here we review available literary data on nuclear distribution of some essential molecular components related to RNA polymerase II-dependent transcription and mRNA processing at different stages of ZGA in mammals. The dynamics of the nucleolar components in mammalian embryos was studied better [20–22] and remains beyond the scope of the present review.

## 2. RNA Polymerase II and Basal Transcription Factors

The association of RNA polymerase II (RNAP II) with many factors to form a polyfunctional holoenzyme is required for transcription [47, 48]. Besides core RNAP II enzyme, this complex includes basal transcription factors, mediator complexes, and transcription coactivators [49–51]. The large RNAP II subunit (RPB1) contains the carboxy-terminal domain (CTD) consisting of multiple heptapeptide repeats with the consensus sequence YSPTSPS. Dynamic phosphorylation of the CTD determines the progression of the RNAP II transcription cycle from initiation through elongation to termination [52–54].

Posttranslational modifications of RNAP II holoenzyme were studied in mouse and rabbit preimplantation embryos with the use of western blot analysis and immunofluorescent microscopy [23]. In this study, a set of the following monoclonal antibodies was applied: 8WG16 revealing the unphosphorylated CTD, CC-3 directed against a phosphorylated epitope of the CTD, and Pol3/3 against an internal epitope distinct from the CTD, thus recognizing two forms of RPB1 simultaneously. The authors have eventually suggested that phosphorylation of the CTD might control ZGA in the embryos of mammals with different ZGA chronologies. Several hours after fertilization, the CTD was found dephosphorylated. Dephosphorylation of the CTD occurs before the onset of a period characterized by a weak transcriptional activity (minor ZGA). Then, the CTD lacks immunological and drug-sensitivity characteristics related to its phosphorylation status, and RNAP II gradually translocates into the nuclei. At the major ZGA phase corresponding the 2-cell stage in mouse and the 8–16-cell stage in rabbit, phosphorylation pattern of the CTD was close to that observed in somatic cells. The authors also reported that actinomycin D does not prevent a new phosphorylation pattern of RNAP II at the onset of the major ZGA. As a result, a phosphorylated embryogenesis-specific RNAP II isoform was identified. This RNAP II isoform is insensitive to DRB, a CTD-kinase inhibitor, since it lacks a phosphoepitope generated by TFIIH-associated kinase phosphorylation. Finally, the authors have concluded that nuclear translocation of RNAP II and CTD phosphorylation might be major determinants of ZGA. The possible association of different RNAP II isoforms with intranuclear structures of mouse and rabbit preimplantation embryos was not explored in detail in this study.

**Figure 1:**
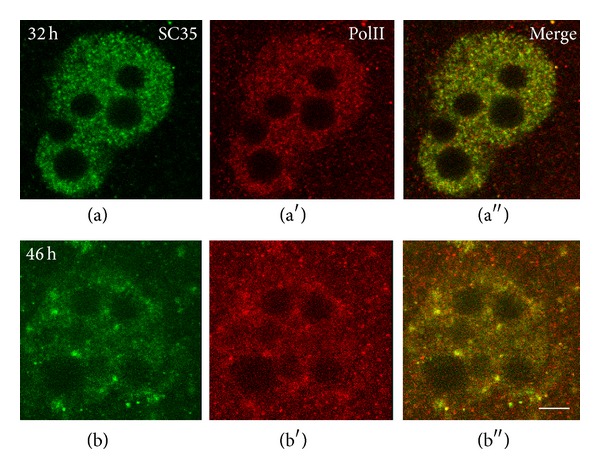
Double immunolocalization of SC35 (a), (b) and hyperphosphorylated RNA polymerase II (PolII) (a′), (b′) in mouse embryos. Fluorescence of nuclei begins to be detected only at the early 2-cell stage (line a). Association of RNAP II with SC35 domains (speckles) is observed already at this stage and is increased when ZGA finishes (line b). Bar is 10 *μ*m, according to [24], open access.

**Figure 2:**
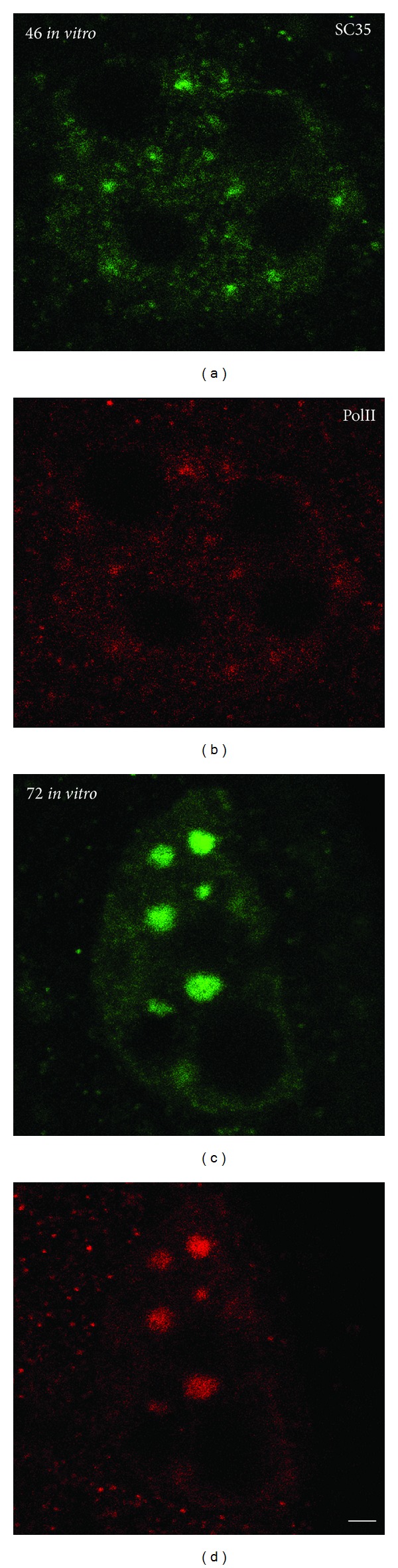
The distribution of SR protein SC35 (a), (c), hyperphosphorylated RNA polymerase II (PolII) (b), (d) in control (a), (b), and arrested* in vitro* 2-cell mouse embryos (c), (d). Note the large SC35 speckles enriched in hyperphosphorylated RNA polymerase II in the nucleus of blocked embryo. Scale bar is 5 *μ*m, according to [26], reprinted from Tissue and Cell; Bogolyubova. Transcriptional activity of nuclei in 2-cell blocked mouse embryos 2011; 43: 262-265 [26], with permission from Elsevier.

In our immunocytochemical experiments, we used an affinity purified polyclonal serum to reveal the hyperphosphorylated form of RNAP II in mouse embryos. This form of RNAP II was detected before the major ZGA phase, namely, in early 2-cell embryos [24]. While ZGA proceeds, RNAP II was found in association with perichromatin fibrils (PFs) [25], which are referred to as the ultrastructural “*in situ* forms” of nascent pre-mRNA transcripts [55]. Besides, the hyperphosphorylated RNAP II was detected in nuclear speckles identified by the presence of the SR protein SC35. The intensity of anti-RNAP II immunostaining in speckles was being increased towards the end of ZGA [24] (Figure 1). If ZGA is delayed, for example, in embryos under the so-called “2-cell block* in vitro*” (for details, see [56, 57]), the hyperphosphorylated form of RNAP II begins to accumulate in enlarged nuclear speckles [26] (Figure 2).

Worrad et al. [29] have explored the concentration of the transcription factors Sp1 and TBP (TATA-binding subunit of TFIID) in 1-cell mouse embryos. The authors showed that concentration of both factors drastically increases in a time-dependent fashion after fertilization during the 1-cell stage. Six hours following the formation of pronuclei, this increase continued, and by the G2 phase the pronuclear concentration of Sp1 and TBP was very similar to that observed in 2-cell embryos. In addition, concentration of both transcription factors was greater in the male pronucleus and the difference was more pronounced for TBP.

Later, TBP and TBP-associated factor 1 (TAF1) were revealed in mouse zygotes [30]. TAF1 was not detected just after fertilization in transcriptionally silent cells, but it is expressed in pronuclei, reaching the maximum before the onset of ZGA. TAF1 and TBP shared similar dynamics of expression patterns. In 4 h after fertilization, anti-TAF1 immunofluorescent signal was not registered. The signal became obvious in a portion of embryos in 6 h after fertilization, in the majority of embryos in 9 h, and in all embryos in 11 h. TBP was revealed in some embryos for the first time in 4 h after fertilization and in almost all pronuclei 1 h later. In male pronuclei, transcriptional activity can be registered earlier. Respectively, both factors initially appear in male rather than in female pronuclei. The authors have supposed that the deficiency of transcription machinery might be a reason for the limitation of transcription at the beginning of embryogenesis.

In addition to TBP, Gazdag et al. [31] have revealed the related protein TBP2, also called TRF3 (TBP-related factor 3), in mouse 1-2-cell embryos. They also reported that TBP is expressed in the oocytes at the beginning of folliculogenesis, but cannot be detected during further stages of oocyte development and becomes abundant again only after fertilization. In contrast to TBP, TBP2 was almost undetectable after fertilization until the 2-cell stage.

**Figure 3:**
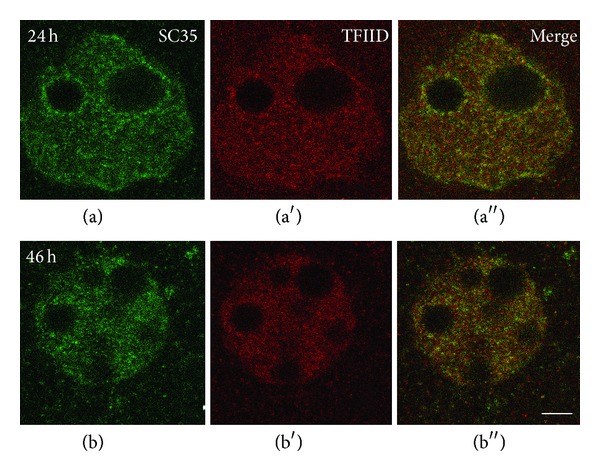
Double immunolocalization of SC35 (a), (b) and transcription factor TFIID (a′), (b′) in mouse embryos. TFIID is revealed in the nuclei at all studied stages. Colocalization of SC35 and TFIID is intensified during ZGA. Bar is 10 *μ*m, according to [24], open access.

In mouse embryo pronuclei, basal transcription factor TFIID has been revealed before the onset of ZGA in association with nuclear speckles already at the zygote stage [24]. During realization of ZGA, the presence of TFIID in the speckles became more evident (Figure 3). Localization data of RNAP II and TFIID in the IGCs of mouse embryos are in accordance with the results that came from somatic cell studies [58, 59] including IGC proteome analysis [60].

Interestingly, TFIID was also clearly detectable at the periphery of the nucleolar precursor bodies (NPBs) at the earliest stages of mouse cleavage [24]. The functional significance of this finding is ambiguous. Further studies are required to clarify the molecular composition and functions of NPBs, enigmatic structures of embryo nuclei.

Synoptic data on the revealing of RNAP II and transcription factors in mammalian embryos are presented in Table 1. At least a portion of RNAP II transcription machinery is detected in mammalian embryo nuclei already before ZGA. However, the final intranuclear patterns of RNAP II and transcription factors are being formed for the whole period of ZGA.

## 3. Pre-mRNA Splicing Factors

Pre-mRNA splicing factors have been detected in the embryos of various mammalian species (Table 1). In the nuclei of early and late 2-cell mouse embryos, the distribution of snRNPs and non-snRNP protein SC35 was studied with the use of immunofluorescent and immunoelectron microscopy [35]. Following the activation of embryonic transcription in late 2-cell mouse embryos, splicing factors are revealed both in IGCs and in association with the PFs.

The Sm antigen of snRNPs and 2,2,7-trimethyl guanosine (TMG) cap characteristic for the mature snRNAs were localized in the nuclei of 1–4-cell porcine embryos at the ultrastructural level [43]. Surprisingly, unlike in bovine embryos (see below), immunoelectron microscopy also revealed the occurrence of snRNPs in the NPBs of porcine embryos. In bovine early preimplantation embryos, snRNPs were localized at different stages of ZGA [40]. Before the onset of transcription, up to the 4-cell stage, a diffuse labeling of the nucleoplasm was revealed. After the beginning of transcription, all 8-cell embryo nuclei were markedly stained, and mRNPs were shown to concentrate at the periphery of chromatin aggregates in association with the PFs. Interestingly,* in vitro*-produced 8-cell embryos showed a higher degree of chromatin condensation and a peripheral distribution of chromatin blocks as compared with the embryos produced* in vivo*. In 2- and 4-cell embryos, an intensive anti-snRNP labeling also characterized IGCs (nuclear speckles), which were often observed in the vicinity of the NPBs or even joined to them at the 4-cell stage. Using antibodies specific for the Sm epitope of snRNPs, the labeling was detected neither in the NPBs during embryonic nucleologenesis nor in the resulting nucleoli.

Analogous studies have been carried out later on bovine and caprine 2-cell embryos [41, 61]. The authors described several types of extrachromosomal nuclear bodies (NBs), 0.2–2.0 *μ*m in diameter. The most striking feature of these NBs was the presence of proteins involved in pre-mRNA splicing. Some NBs having a rather dense finely fibrillar composition and, thus, named the dense bodies (DBs) were shown to contain the Sm-antigen. Moreover, more numerous NBs differed morphologically from the former by a much looser composition of fibrillogranular elements (called loose bodies, LBs). This type of the NBs, in addition to the Sm-antigen, contained the non-snRNP splicing factor SC35, a marker of IGCs/speckles. Both types of the NBs were distinguished clearly from the NPBs both morphologically and by the absence of NPB immunolabeling with antibodies against pre-mRNA splicing factors. Apart from the NBs described above, a high concentration of snRNPs was revealed in rather small, approximately 0.05 *μ*m in diameter, morphologically and poorly defined domains named small snRNP-enriched areas (SSA). These domains housed a set of nuclear proteins typical for the CBs, including the CB signature protein coilin. However, it is still elusive whether the LBs correspond to the canonical IGCs of other cells. In the same way, it is unknown whether the DBs or SSA resemble the CBs in all respects.

With use of different approaches including light and electron immunocytochemistry and* in situ* hybridization, the dynamics of the CBs and/or IGCs has been studied in the early embryos of the mouse [24, 34, 62] and the golden hamster [44]. In the mouse, the CBs are already present in 1-cell embryos, before major transcription activation. On the contrary, in hamster embryos they appear during the 2-cell stage after the onset of transcription. On the other hand, hamster 1-cell embryos already display prominent IGCs, whereas typical IGCs appear only at the 2-cell stage in the mouse. Thus, the assembly of both CBs and IGCs seems to be independent of the onset of transcriptional activity in the early mammalian embryo [44]. Zatsepina et al. [62] have shown in early mouse embryos heterogeneity of CB-like (coilin-containing) nuclear bodies both in size and molecular composition. So, anti-Sm antibody was shown to stain small coilin-positive foci present in 1-cell and early 2-cell embryos and the periphery of significantly larger bodies present in middle and late 2-cell embryos.

Little is known about mammalian embryo nuclear structures that virtually could correspond to perichromatin granules (PGs). It is suggested that the structure of PGs arises due to the packaging of PFs, and the PGs participate in transient storage of mRNPs (in the form of heterogeneous nuclear (hn) RNPs) and/or also in mRNP nucleoplasmic transport [63]. Typical mammalian somatic PGs contain some splicing snRNAs [64] and snRNP proteins [65].

Numerous small PG-like granules, approximately 150 nm in diameter, resembling similar structures in the nucleus of antral oocytes [66] have been described in the nuclei of mouse 2-cell embryos [9]. Since the 4-cell stage, the number of the PGs decreases in the nuclei of mouse blastomeres and becomes comparable with their number in somatic cells. Taking into account the gradual increasing of embryonic RNA synthesis at this period, the authors believe that at least a part of the PGs in 2-cell mouse embryos contain maternal hnRNPs that will migrate into the cytoplasm or degrade. It cannot be excluded that some RNAs present in the PGs of mouse embryos may play a regulatory role.

The nature of larger RNP-containing granules, 30–50 nm in diameter, that have been described in the nuclei of 2–8-cell mouse embryos [9] remains unknown.

## 4. Some Proteins of the Exon-Exon Junction Complex

In our group, we also studied nuclear distribution of some representative proteins constituting the exon-exon junction complex (EJC) [45, 46, 67]. The EJC is a multiprotein complex that is loaded onto mRNA during splicing at a precise position upstream of exon-exon junctions [68]. Amongst other functions, the EJC enhances nuclear export of mRNA providing a platform to bind export factors [69]. Core EJC proteins including Y14 [70] provide stable association of the EJC with mRNA, whereas EJC shell proteins (e.g., Aly/REF) serve as adaptors providing a link between splicing and export [71].

In the nuclei of mouse embryos at the different stages of ZGA, the EJC core protein Y14, the shell protein Aly, and the essential mRNA export factor NXF1/TAP were detected in transcriptionally inert 1-cell embryos, but the intensity of fluorescence increased in late 2-cell embryos that are transcriptionally active [67]. Possible association of NXF1/TAP with nuclear speckles was also studied at the 2-cell stage [45]. In transcriptionally active embryos, NXF1/TAP was detected in the vicinity of the IGCs/speckles rather than inside these nuclear domains. Artificial inhibition of transcription by drugs resulted in significant accumulation of NXF1 in enlarged speckles.

We also studied possible spatial interactions between the EJC proteins and nuclear actin with the use of Förster resonance energy transfer (FRET) [46]. Two patterns of the FRET signal were detected in transcriptionally active nuclei of 2-cell embryos for the pairs: actin-Y14, actin-Aly/REF, and actin-NXF1/TAP. FRET areas were revealed both randomly distributed in the nucleoplasm and in association with NPBs. The means of FRET efficiency exceeded 25%–30% in separate areas. We supposed that FRET signals in the nucleoplasm correspond to transcriptionally active chromatin zones. The presence of Y14 and NXF1/TAP in the NPBs was confirmed at the ultrastructural level [46]; however, it is hard to explain detection of FRET in these nuclear structures of the embryos. The FRET pattern, typical for transcriptionally active embryos, was being retained after artificial suppression of transcription by drugs. In the specimens digested by RNAse, FRET efficiency was decreased significantly, showing the spatial interactions between EJC proteins and actin occur in an RNA-dependent manner.

## 5. Closing Remarks

The nucleus of early mammalian embryos is a highly dynamic system. The bulk of studies were devoted to the dynamics of intranuclear accumulation of the revealed antigens but not to their association with nuclear domains. Available data on the domain organization of mammalian embryo nuclei are not comprehensive, except for nucleolus; the genesis of the CBs and IGCs/speckles was described in the mouse and the golden hamster. As concerns the embryos of farm animals, real relationships between multifarious nuclear bodies known under various names and the canonical nuclear domains of somatic cell have to be ascertained. High rates of nuclear structure reorganization together with different chronologies that characterize early embryogenesis in various organisms make a comparative analysis of embryo nuclear domains difficult. Further studies in this field would be helpful to form an integral concept of the structural and functional compartmentalization of the nucleus as well as to extend our knowledge on the mechanisms of early embryogenesis in mammals.

## Figures and Tables

**Table 1:** RNA polymerase II, mRNA processing factors, and mRNA export factors revealed in early mammalian embryos.

Group of factors	Animal	Detected antigen	Method	Stage of embryogenesis	Reference
RNAP II	Mouse, rabbit	core of the RPB1 RNAPIIa, RNAPIIo	IF*, WB	1-cell–16–32 cell	[23]
	Mouse	RNAPIIa, RNAPIIo	IF, IEM	1-cell-2-cell	[24–27]

mRNA transcription factors	Mouse	TBP	IF	1-cell	[28]
		Sp1, TBP	IF Microinjection of Sp1-dependent luciferase reporter gene	1-cell-2-cell	[29]
		TBP, TAF1	IF	1-cell	[30]
		TBP, TBP2	IF, WB	1-cell-2-cell	[31]
		TFIID	IF	1-cell-2-cell	[24]

Splicing factors	Mouse	U1, U2, U4, U6 snRNAs snRNPs	ISH IF	1-cell-blastocyst	[32]
		U1 snRNA U1 snRNP	ISH IF	1-cell-blastocyst	[33]
		U1, U2 snRNAs snRNPs, SC35	ISH IF	1-cell–8-cell	[34]
		snRNPs, SC35	IF, IEM	1 cell-2-cell	[24, 35, 36]
		snRNPs	IF	1-cell-2-cell	[27]
		snRNPs	IEM	2-cell	[37]
		hnRNPs, snRNPs	IEM	1-cell–8-cell	[38]
	Bovine	U2 snRNA snRNPs	ISH, NB IF	1-cell-blastocyst	[39]
		snRNPs, SC35	IEM	1-cell–16-cell	[40, 41]
	Caprine	snRNPs, SC35	IEM	1-cell-2-cell	[41]
	Porcine	snRNPs	IF	1-cell-blastocyst	[42]
		snRNPs, SC35	IEM	1-cell–4-cell	[43]
	Hamster	U1, U2 snRNAs snRNPs SC35	ISH IF IF, IEM	1-cell–8-cell	[44]

mRNA export-related factors	Mouse	hnRNPs	IF, WB	1-cell–8-cell 1-cell	[34] [28]
		hnRNPs, Y14, Aly/REF, NXF1/TAP	IF, IEM	1-cell-2-cell	[45, 46]
	Hamster	hnRNPs	IF	1-cell–8-cell	[44]

*Abbreviations: IF: immunofluorescence; IEM: immunoelectron microscopy; ISH: *in situ* hybridization; NB: northern blotting; RNAPIIa: hypophosphorylated form of RNA polymerase II; RNAPIIo: hyperphosphorylated form of RNA polymerase II; WB: western blotting.
